# Peri-anastomotic microdialysis lactate assessment after esophagectomy

**DOI:** 10.1007/s10388-021-00846-w

**Published:** 2021-05-29

**Authors:** Jakob Hedberg, Gustav Linder, Magnus Sundbom

**Affiliations:** Department of Surgical Sciences, Uppsala University, 75185 Akademiska Sjukhuset, Uppsala, Sweden

**Keywords:** Esophagus, Microdialysis, Anastomotic leak

## Abstract

**Background:**

Esophagectomy is the cornerstone in curative treatment for esophageal and gastroesophageal junctional cancer. Esophageal resection is an advanced procedure with many complications, whereof anastomotic leak is the most dreaded. This study aimed to monitor the microcirculation with microdialysis analysis of local lactate levels in real-time on both sides of the esophagogastric anastomosis in totally minimally invasive Ivor-Lewis esophagectomy.

**Materials and Methods:**

Twenty-five patients planned for esophageal resection with gastric conduit reconstruction and intrathoracic anastomosis were recruited. A sampling device, the OnZurf^®^ Probe, along with the CliniSenz^®^ Analyser (Senzime AB, Uppsala Sweden) was utilized for measurements. Lactate levels from both sides of the anastomosis were analysed in real time, on site, by a transportable analyser device. Measurements were made every 30 min during the first 24 h, and thereafter every 2 hours for up to 4 days.

**Results:**

All probes could be positioned as planned and on the third postoperative day 19/25 and 15/25 of the esophageal and gastric probes, respectively, continued to deliver measurements. In total, 89.6% (1539/1718) and 72.4% (1098/1516) of the measurements were deemed successful. The average lactate level on the esophageal side of the anastomosis and the gastric conduit ranged between 1.1–11.5 and 0.8–7.0 mM, respectively. Two anastomotic leaks occurred, one of which had persisting high lactate levels on the gastric side of the anastomosis.

**Conclusion:**

Application and use of the novel CliniSenz^®^ analyser system, in combination with the OnZurf^®^ Probe was feasible and safe. Continuous monitoring of analytes from the perianastomotic area has the potential to improve care after esophageal resection.

## Introduction

Esophageal cancer and cancer of the gastroesophageal junction (Siewert I and II) is the eight most common type of cancer globally and the incidence rate is rising [1, 2]. The poor overall 5-year survival of around 10% makes esophageal cancer the seventh cancer globally ranked by years of life lost [3]. For localized disease, surgical resection, as part of multimodal treatment, provides the best chance of cure. Esophagectomy is, however, one of the most demanding procedures of gastrointestinal surgery, with up to six per cent 90-day mortality, even after centralization to expert centres [4], and anastomotic leakages in 11–30% of cases [5–7]. In addition to being a life-threatening complication, leakage of the esophagogastric anastomosis is associated with dire consequences for the patient in terms of prolonged length of stay and subsequent reduced quality of life [8, 9] as well as increased health-care costs [10].

Anastomotic leak can be the result of technical error in anastomotic construction or caused by impaired healing, the latter considered to be a result of compromised perfusion of the anastomosis. Several attempts to assess the perfusion in the gastric conduit have been made using pulse oximetry [11], doppler flowmetry [12] as well as tonometry [13]. All these have, however, been focused on the gastric side of the anastomosis where ischaemia, as a result of devascularisation of the gastric conduit, is likely to play an important role.

Microdialysis is a method for measuring analytes in target organs and has been widely used in neurosurgery [14] as well as for evaluation of solid organ perfusion in transplant settings [15]. One historical draw-back of microdialysis sampling has been the need to enter the parenchyma of the organ of interest in order to perform the analysis. To address this issue, a new device, the OnZurf^®^ probe (Senzime AB, Uppsala, Sweden), has been developed. The principle of using surface dialysate has been validated on the small bowel [16], liver [17] and the ischemic heart [18] in experimental settings*.* The present study aimed to monitor the microcirculation in real-time on both sides of the esophagogastric anastomosis in totally minimally invasive Ivor-Lewis esophagectomy.

## Materials and methods

All patients planned for esophageal resection with gastric conduit reconstruction and intrathoracic anastomosis at a tertiary referral centre were invited to participate in this pilot study.

### Microdialysis catheter and analysis system

A sampling device, the OnZurf^®^ Probe, a 150-cm-long microdialysis catheter equipped with a 15 mm dialyse membrane with a pore size of 10 kDa, along with the CliniSenz^®^ Analyser (Senzime AB, Uppsala Sweden) was utilized for measurements. The analyser featured an enzyme-based heat flow detection device incorporated on a microfluidic chip, which aided in avoiding substance interferences. The system enabled continuous and fast sampling of small volumes of either lactate, glucose or pyruvate, the first of which was analysed in the present study.

### Surgical procedure and application of microdialysis probes

All operations were performed as minimally invasive two-stage procedures. A standard gastric mobilization and abdominal lymphadenectomy was performed in the supine position and the gastric conduit was constructed 4–5 cm wide by multiple firings of linear staplers from the incisura angularis to the top of the fundic dome. The right gastro-epiploic arcade and the right gastric artery were preserved. The patient was then placed in the prone position. After mobilization of the esophagus and completion of the thoracic lymphadenectomy, the specimen was resected and the gastric conduit pulled up to the level of the anastomosis. A side-to-side esophagogastric anastomosis was constructed with a 45-mm linear staple cartridge and hand-sewn closure of the remaining defect and subsequently wrapped with omentum. Finally, one 28-Fr active chest drain was introduced.

**Fig. 1:**
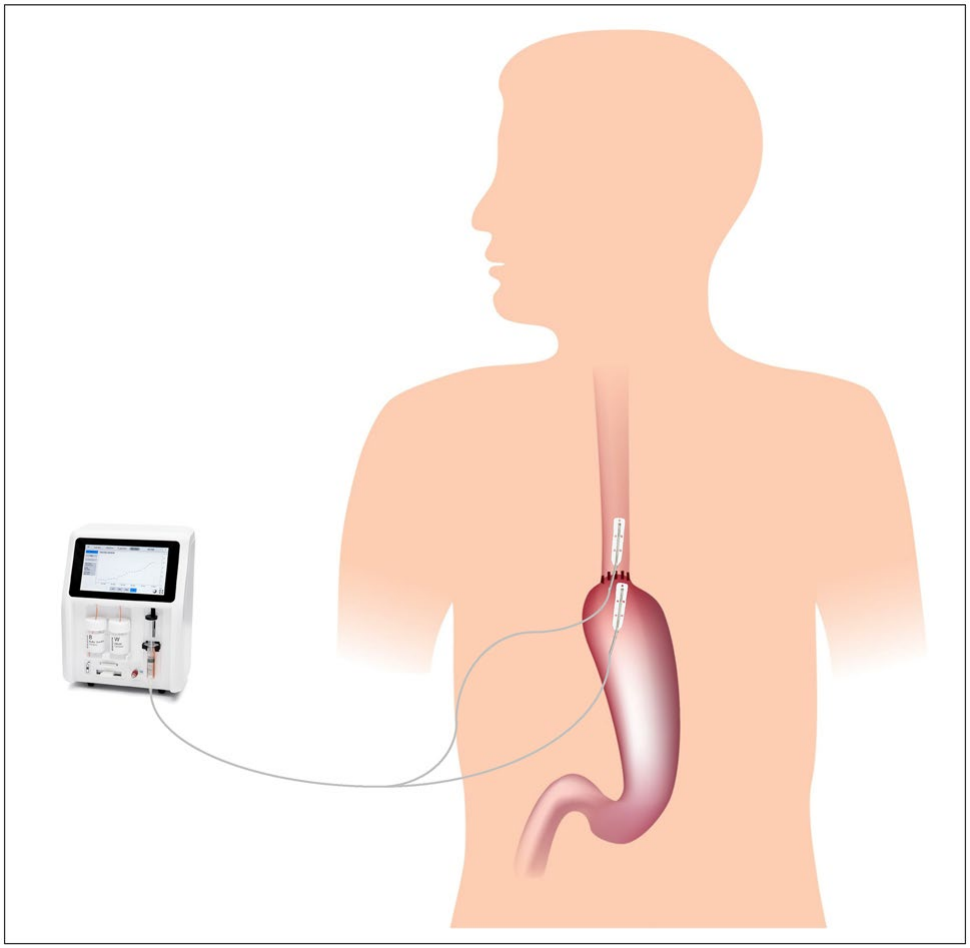
Placement of the esophageal and gastric probes. Note that the esophageal and gastric probes were fixated under the mediastinal pleura and omental wrap, respectively, and attached to separate analysers

Two OnZurf^®^ probes were introduced through an existing trocar and one of the probes was secured on the proximal side of the anastomosis between the esophagus and pleura. A second probe was secured on the serosal surface of the tip of the gastric conduit under the omental wrap. The probes were tested for functionality before and after placement in the thoracic cavity. In the first 8 cases, the probes were externalized along with, and taped to, the chest drains and in the subsequent 17 cases, they were passed through a separate trocar incision. Post procedure, all patients were extubated in theatre and upon arrival to the high-dependency unit, the two probes were connected to one CliniSenz^®^ Analyser instrument each (Fig. 1).

### Measurement procedure

Systemic lactate levels were recorded during care at the high dependency unit. Lactate levels in the dialysates were analysed in real-time on site by the transportable analyser device. Measurements were made every 30 min during the first 24 h, after which the patients were transferred from the high dependency unit to ward level care. Following re-calibration of the CliniSenz^®^ system, measurements were performed every 2 hours for up to 5 days. To facilitate postoperative mobilization, the two measuring devices were placed on a specially constructed and adjustable support walker (cardiac walker), also equipped with battery back-up. Staff were instructed to remove the probes from the measuring devices in case of an emergency. Following a routine chest CT with intravenous and peroral contrast on day 5, the probes were removed. CT-scans were scrutinized for anastomotic leak, pneumonia, pneumothorax (< 1, 1–2 and > 2), and subcutaneous emphysema (negligible, minor or moderate amount, i.e., visible air in the neck, arm or along the whole thoracic wall).

All lactate measurements were blinded to clinical personnel, and thus not used in the clinical management of patients. The postoperative lactate microdialysate measurements were considered successful if being within the prespecified range of 0.5–12 mM.

### Statistics

No sample size calculation was performed due to the exploratory nature of the study. Data regarding clinical characteristics are presented as median with range. Average and standard error of lactate levels are presented as well as percentage of successful measurements.

Ethical statement: The study was conducted according to the guidelines of the declaration of Helsinki and approved by the Regional Ethics committee in Uppsala (Dnr 2016/562).

## Results

Twenty-five patients were included in the study, Table 1. All operations were completed with a minimally invasive approach with a median operating time of 264 min (range 215–454) and a median blood loss of 100 ml (range 0–300 ml), Table 2. Attempts to suture the device with 5-0 Vicryl^®^ (Ethicon, Cincinnati, OH, USA) to the gastric conduit and esophagus were abandoned after the first patient due to technical issues and instead placement under the pleura and omental wrap, respectively, was employed.

**Table 1:** Patient and tumor characteristics of 25 included patients undergoing minimally invasive esophagectomy for esophageal cancer

Patient characteristics	
Age, median (range)	
Years	71 (53–81)
Gender, no (%)	
Woman	2 (8%)
Man	23 (92%)
Weight, median (range)	
Kg	74 (55–126)
BMI, median (range)	
kg/m^2^	25 (18–38)
Tumor characteristics	
Tumor location, no (%)	
Middle esophagus	2 (8%)
Distal esophagus	16 (64%)
Gastroesophageal junction	7 (28%)
Histology, no (%)	
Adenocacinoma	23 (92%)
Squamous cell carcinoma	2 (8%)
Tumor stage, no (%)	
I a–b	8 (32%)
II a–b	6 (24%)
III a–c	11 (44%)
IV	0 (0%)
Neoadjuvant treatment, no (%)	
None	4 (16%)
Chemotherapy	12 (48%)
Chemoradiotherapy	9 (36%)

**Table 2:** Peroperative data and postoperative results in the 25 patients following minimally invasive esophagectomy with gastric tube reconstruction and intrathoracic anastomosis

Peroperative data	
Duration of surgery, median (range)	
min	264 (215–454)
Blood loss, median (range)	
ml	100 (0–300)
Externalisation of microdialysis catheter, no (%)	
With chest tube	8 (32%)
Separate incision	17 (68%)
Postoperative data	
Radiology findings day 5 (CT, *n* = 24)	
Leakage of peroral contrast	0 (0%)
Pneumonia	1 (4%)
Pneumothorax	13 (54%)
< 1 cm	10 (42%)
1–2 cm	2 (8%)
> 2 cm	1 (4%)
Surgical emphysema	
Small amount	13 (54%)
Minor	7 (29%)
Moderate	2 (8%)
Major postoperative complications, no (%)	
Clinical anastomotic leak	2 (8%)
Respiratory complications	3 (12%)
Cardiovascular complications	2 (8%)
Reoperation	0 (0%)
Death	0 (0%)
Length of stay, median (range)	
Days	10 (8–55)

### Lactate readings

**Fig. 2:**
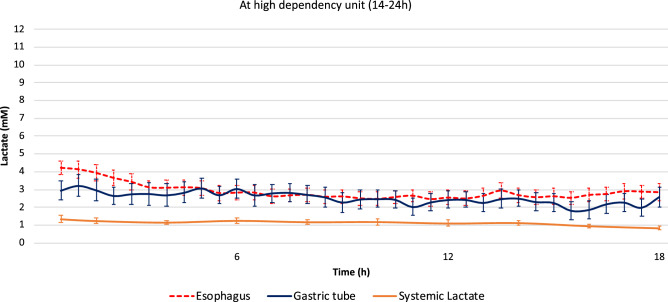
Lactate levels during the overnight stay at the high dependency unit. Average and standard error of the mean are presented. The orange line represents systemic lactate levels from blood gas analysis at the high dependency unit

Patients stayed 18 (14–24) hours in the high dependency unit. Systemic lactate levels were generally low, with a mean of 1.1 and a standard deviation of 0.5 mM. All, but two of the gastric probes (#9 and #25), started to deliver measurements when the system was activated. In retrospect, we believe that the two non-functioning probes were accidentally damaged when closing the incision site at the end of the procedure. In total, 89.8% (886/987) and 71.1% (604/850) of the half-hourly measurements were within the prespecified range from the esophageal and gastric probes, respectively. In individual patients, the average lactate level on the esophageal side of the anastomosis and the gastric conduit ranged between 1.1–8.0 and 0.8–5.3 mM, respectively. The measurements over time are demonstrated in Fig. 2.

**Fig. 3:**
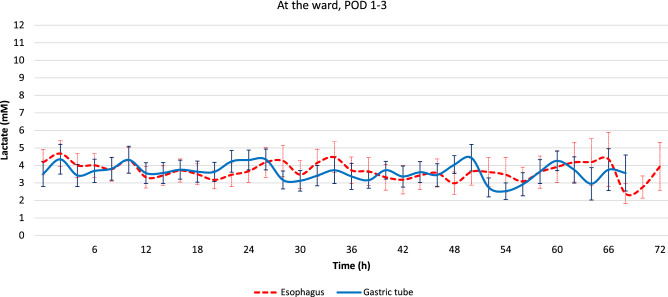
Lactate levels at the surgical ward, i.e., postoperative day 1 to 3, after re-calibration of the system. In the morning of the third postoperative day (here after 48 h), 76% (19/25) and 60% (15/25) of the esophageal and gastric probes, respectively, continued to deliver measurements. Average and standard error of the mean are presented

Successful measurements at the ward were achieved during a period of 24–76 h. In 89.3% (653/731) and 74.2% (494/666) of these, the lactate levels were within the prespecified range, and ranging between 1.1–11.5 and 1.3–7.0 for the esophageal and gastric probe, respectively. On the morning of the third postoperative day (> 48 h), 76% (19/25) and 60% (15/25) of the esophageal and gastric probes, respectively, continued to deliver measurements. Figure 3 demonstrates the mean lactate levels during the first 3 postoperative days.

The rate of successful measurements was 82% overall, however, the functionality of the esophageal probe seemed superior (89 vs. 72% successful measurements, *p* < 0.001).

**Fig. 4:**
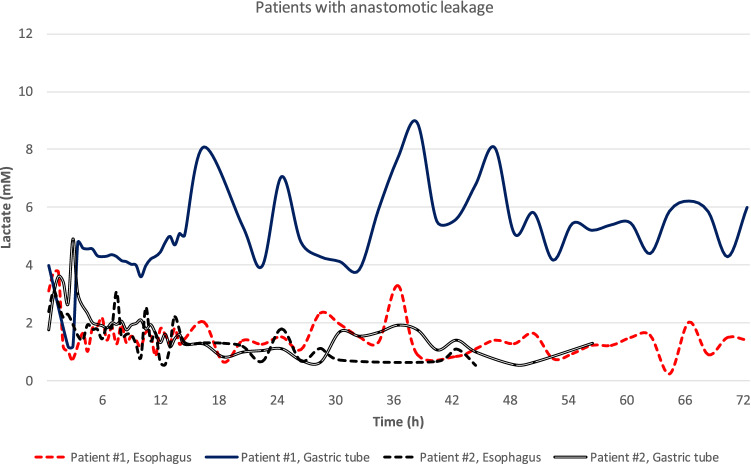
Lactate levels in the two patients with anastomotic leakage. In patient #1, a persisting high lactate level was seen in the gastric tube (), visible already during the first postoperative hours. Note the marked increase in lactate in the gastric tube of this patient compared to the remaining measurements illustrated in Figs. 2 and 3

Out of all included patients, two had an anastomotic leak (2/25, 8%). Lactate curves for these patients were plotted separately and one revealed persisting high surface lactate levels from the gastric conduit starting 4 h postoperatively (Fig. 4). This patient had a normal initial postoperative course, except for two short episodes of fever and moderate increased oxygen demand, as well as an unremarkable CT-scan with peroral contrast. The leak became clinically evident on postoperative day 6.

### Radiological findings

On the routine CT, performed on postoperative day 5, 13 out of 25 patients had surgical emphysema, of which 7 a negligible amount, 4 a minor amount and 2 patients moderate surgical emphysema. Both cases of moderate surgical emphysema occurred in the first part of the series when the probes were externalized alongside the chest tube. Pneumothorax was present in 13 patients (less than 1 cm apical in 10 patients, 1–2 cm in 2 patients and more than 2 cm in 1 patient). One patient had radiological signs of pneumonia.

### Removal of probes and clinical data

No emergencies requiring urgent removal of the probes from the measuring devices occurred. All probes could easily be removed as planned, without pain or any other immediate complications. The median length of stay was 10 days (range 8–55) and no 90-day mortality was observed. At follow-up, no local problems were noted at the externalization site of the probes.

## Discussion

Application of the OnZurf^®^-probe and direct analysis of lactate levels on both sides of the esophagogastric anastomosis was feasible and safe. In one out of two patients with anastomotic leak, high lactate levels, persisting throughout the measurements, were seen. Externalizing the probes through a separate incision seemed to reduce the risk for subcutaneous emphysema in absolute numbers.

### The studied patients

The clinical characteristics of the 25 studied patients (71 years old in mean, 92% men and a dominance of adenocarcinoma and neoadjuvant treatment) are in line with esophageal cancer care in Sweden [4]. The operative time was not excessively prolonged by placing the microdialysis catheters, here 264 min in comparison to a mean of 300 min in our national quality registry. Neither the rate of anastomotic leakages (8%) nor the length of stay surpassed recently published series [19, 20], although this study was not powered for detecting such differences.

### The use of microdialysis

Traditionally, microdialysis measurements have been performed in a more invasive manner, such as intracranial microdialysis in traumatic brain injury [21, 22]. In investigations of the peri-anastomotic region after esophagectomy, invasive measurements are not practical since they would require weakening the anastomotic region by placing a microdialysis catheter in the esophageal and/or gastric wall, thus damaging this delicate region. The present study instead utilized a microdialysis probe measuring lactate from the surface of the investigated organ. Previous experimental studies have shown that surface derived lactate is comparable to intraparenchymally derived lactate [18]. Furthermore, utilizing a plastic covered surface microdialysis probe allows detection of local metabolic changes earlier than do intraparenchymatous probes[17] thus allowing for studies, where intra organ measurements are not feasible. In a study from 2016, Åkesson et al., demonstrated that a plastic covered microdialysis probe, as used in the present study, allowed for detection and monitoring of small bowel ischemia from 20 min after its onset [16]. Even though clinical ischemia can be detected and monitored with surface microdialysis of lactate, the important question of what cut-off value regarding lactate is clinically relevant still remains to be answered. Any lactate value above the systemic lactate would suggest increased anaerobic metabolism in the organ of interest. Difficulties arise in interpreting microdialysis derived lactate if patients become ill for other reasons than anastomotic leakage and subsequently develops systemic acidosis with any degree of systemic lactatemia. In this study, the systemic lactate was consistently low (below 2 mM) and the microdialysis fluctuations are not likely to be caused by variations in systemic lactate levels. In a Danish study from 2014 using free mediastinal microdialysis in 60 patients undergoing esophagectomy, the authors could create a mathematical model using a number of microdialysate metabolites to predict early anastomotic leaks before they became clinically detectable [23]. Further, higher mean lactate values and a greater AUC for lactate measurements was seen in patients with anastomotic complications compared to patients without complications. In similarity with the present study, however, it was not feasible to detect a cut off value for peri anastomotic lactate although it is noted that the mean values of lactate were similar to the findings of the present study. Continuous correlation of microdialysis derived lactate to systemic lactate could possibly further clarify this matter and should be the focus of further studies.

### The measuring system in clinical practice

The proximal probe was placed in a pocket in-between the mediastinal pleura and the esophageal wall and the gastric probe was placed under the omental wrap, without being fixated by sutures. The superior functionality of the esophageal probe might be a result of the snug fixation between the unopened mediastinal pleura and the untouched proximal esophagus.

At the surgical ward, postoperative mobilization was not hindered by the probes to any greater extent. However, an increased catheter length would have been beneficial for increased mobility. The dedicated and specially constructed support walker worked adequately as well as the routine charging of the battery unit. However, a smaller battery-powered analysis unit would have facilitated the daily care of the patient at the surgical ward. Finally, the probes were not radiopaque enough to be visible on the postoperative routine CT-scans, a draw-back which should be corrected before launching the system into clinical practice.

Although many authors describe the need for both lactate, glucose and pyruvate as well as various quotients of these variables, we could demonstrate high surface lactate values already during the first postoperative hours in one of the two patients having an anastomotic leak. Interestingly, the patient with high lactate levels in the gastric tube had an otherwise uneventful recovery the first postoperative days, consistent with the leak being a delayed result of early ischemia. Thus, this monitoring system has unique potential for detection, correction and follow-up of organ ischemia in the postoperative setting and might guide measures to improve oxygenation, rheology, end organ perfusion and other optimizable factors.

### Strengths and limitations

Among the strengths of the present study is the direct collaboration with the manufacturer, being present in the operation theatre for the first procedures and setting up the CliniSenz^®^ analyser in all patients. Also, the typical characteristics of the included patients, representing an average patient cohort with good postoperative outcome increase generalizability. A limitation is the sample size of the study, as there were not enough anastomotic leakages to fully evaluate the prognostic value of the system. Moreover, the choice not to suture the probes to the serosa of the studied organs could have increased the risk of catheter dislodgement, thus explaining some of the lacking measurements.

In summary, the use of the novel CliniSenz^®^ analyser system, in combination with the OnZurf^®^ Probe, was feasible and resulted in firsthand clinical experience and several conceivable improvements. Most important, we are approaching the possibility of continuous evaluation of a high-risk intrathoracic anastomosis by an easy-to-use patient-monitoring system.
